# Thyroid Fibrosarcoma as a Rare Differential Diagnosis of Anaplastic Thyroid Cancer: A Case Report

**DOI:** 10.1155/crie/6675175

**Published:** 2025-08-19

**Authors:** Phichaya Chamnanvej, Bantita Phruttinarakorn, Rattanakan Chaiprasithikul, Nuttapong Topibulpong, Chutintorn Sriphrapradang

**Affiliations:** ^1^Department of Medicine, Somdech Phra Debaratana Medical Center, Faculty of Medicine Ramathibodi Hospital, Mahidol University, Bangkok 10400, Thailand; ^2^Department of Pathology, Faculty of Medicine Ramathibodi Hospital, Mahidol University, Bangkok 10400, Thailand; ^3^Department of Medicine, Faculty of Medicine Ramathibodi Hospital, Mahidol University, Bangkok 10400, Thailand

**Keywords:** case report, connective tissue neoplasms, head and neck cancer, kinase inhibitors, precision medicine, thyroid neoplasm

## Abstract

**Introduction:** Thyroid gland fibrosarcomas are very rare tumors, with only very few cases have been reported in the literature. Their similarity to anaplastic thyroid cancer poses a diagnostic challenge, often leading to misdiagnosis.

**Case Report:** We report the case of an 87-year-old female with a history of left thyroid nodule who underwent a left lobectomy and subsequently, received levothyroxine therapy. She presented with a rapidly growing mass on the right thyroid gland. Her thyroid function was normal. Ultrasound revealed an ill-defined hypoechoic mass measuring 4 cm on the right thyroid. Fine-needle aspiration biopsy (FNAB) was performed, and cytology indicated Bethesda VI for anaplastic thyroid carcinoma. After the total thyroidectomy, the surgical pathological examination revealed a high-grade fibrosarcoma with extension into the strap muscle. Lymphovascular and perineural invasion was noted. Immunohistochemical staining showed positivity for smooth muscle actin (SMA), and negative for paired-box gene 8 (PAX8), transcription factor 1 (TTF-1), thyroglobulin, and epithelium markers (AE1/AE3). Following surgery, adjuvant therapy with radiation and chemotherapy using ifosfamide was administered. However, the disease progressed with lung metastasis. The treatment was changed to administration of pazopanib, resulting in dramatic improvement of lung metastasis. However, the disease continued to progress, and patient passed away within 2 years after treatment initiation.

**Conclusions:** Although fibrosarcoma of the thyroid gland is exceedingly rare, it should be considered in the differential diagnosis of anaplastic thyroid carcinoma. Immunohistochemistry (IHC) plays a crucial role in supporting the diagnosis. A multidisciplinary approach is essential for its management. In addition to surgery, emerging adjuvant therapies with kinase inhibitors have shown promise in improving patient survival.

## 1. Introduction

Thyroid cancer is one of the most common malignancies, with papillary thyroid carcinoma being the most prevalent subtype of well-differentiated thyroid cancer. This subtype is generally recognized for its indolent course and favorable prognosis [1, 2]. However, rarer and more aggressive subtypes, such as differentiated high-grade thyroid carcinoma, poorly differentiated thyroid carcinoma, and anaplastic thyroid carcinoma, are also encountered [3]. Additionally, different types of malignant tumors, including nonfollicular cell-derived neoplasms, can pose diagnostic challenges within the context of thyroid neoplasms.

Primary thyroid sarcoma is an extremely rare malignancy, accounting for only 0.01%–1.5% of all thyroid cancers [4]. It typically affects older adults, with the highest incidence reported in individuals aged 60–79 years [4]. Histologically, thyroid sarcomas encompass diverse subtypes, including angiosarcoma, malignant hemangioendothelioma, malignant fibrous histiocytoma, leiomyosarcoma, and fibrosarcoma. Due to its aggressive nature and overlapping clinical and imaging features with other thyroid cancers, thyroid sarcoma is often misdiagnosed, resulting in delayed or inappropriate treatment.

This case report describes, a rare case of thyroid fibrosarcoma that was initially misdiagnosed as anaplastic thyroid carcinoma. This emphasizes the critical need for a high index of suspicion and comprehensive histological evaluation, including immunohistochemistry (IHC), to achieve an accurate diagnosis of these aggressive thyroid neoplasms. Given the rarity of thyroid sarcomas, a multidisciplinary approach involving experienced pathologists, radiologists, and clinicians is essential to ensure optimal patient management.

## 2. Case Presentation

An 87-year-old woman with a history of type 2 diabetes mellitus, hypertension, coronary artery disease, and a left lobectomy for a benign thyroid nodule over 30 years ago presented with a rapidly enlarging mass in the right thyroid. She had been on levothyroxine replacement therapy following the lobectomy. Examination revealed a firm, fixed 1.5 cm nodule in the right thyroid lobe without cervical lymphadenopathy.

**Figure 1:**
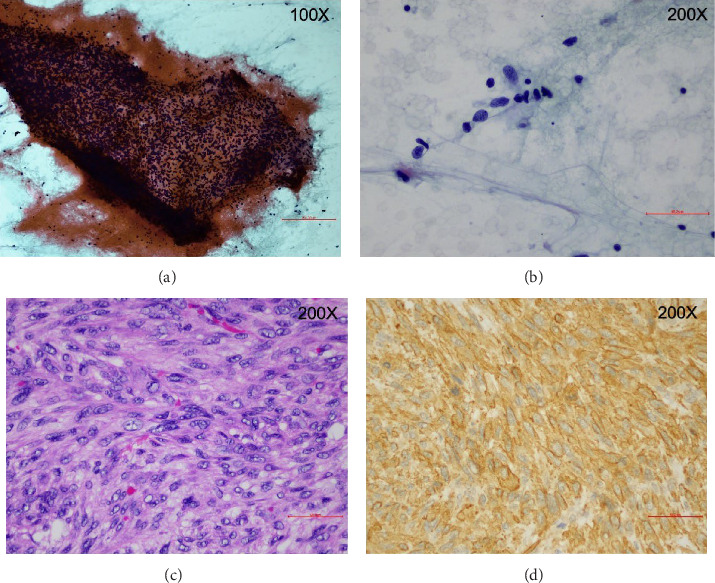
Right thyroid mass. Cytology: (A) Papanicolaou stain, 100×: loosely cohesive group of neoplastic cells with spindle shape; (B) Papanicolaou stain, 200×: single neoplastic cells with spindle shape contain hyperchromatic nuclei. Pathology: (C) H&E stain, 200×: atypical spindle cells are arranged in a short fascicle; and (D) immunohistochemistry, 200×: positive staining for smooth muscle actin (SMA) in tumor cells.

**Figure 2:**
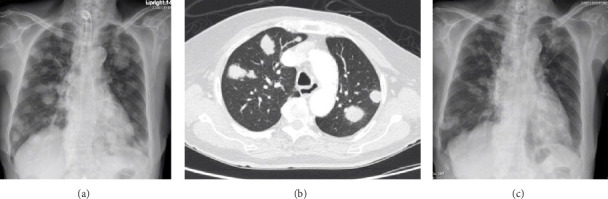
Chest imaging. (A) Chest X-ray: multiple pulmonary nodules of varying sizes in both lungs; (B) CT chest: multiple pulmonary nodules and subpleural masses/nodules in both lungs, ranging from 0.3 to 3 cm in size; and (C) chest X-ray after treatment with pazopanib: decreasing in size of all multiple pulmonary nodules and masses.

Laboratory investigations showed normal thyroid function. Ultrasound of the thyroid gland showed a 3.1 cm × 3.67 cm × 3.69 cm hypoechoic, irregular mass with microcalcifications on the right side and no residual thyroid tissue in the left thyroid bed. Fine-needle aspiration biopsy (FNAB) of the mass (Figure 1A,B) revealed scattered single and loose groups of neoplastic cells with oval to irregular hyperchromatic spindle to pleomorphic nuclei, and scant granular to dense cytoplasm, leading to a preliminary diagnosis of anaplastic thyroid carcinoma (Bethesda category VI: malignant) [5]. A chest X-ray (Figure 2A) and computed tomography (CT) scan of the chest (Figure 2B) demonstrated multiple pulmonary nodules, with the largest measuring 0.5 cm.

A total thyroidectomy was performed. Intraoperative findings revealed extensive invasion of the right thyroid mass involving the trachea and the recurrent laryngeal nerve, necessitating tracheal resection and repair with tracheostomy. Postoperative complications included subcutaneous emphysema and dyspnea.

The surgical specimen consisted of a 5 cm × 5 cm × 3.4 cm thyroid gland, weighing 54.6 g. Gross examination revealed a 4.7 cm ill-defined mass with a rubbery to firm tan-yellow appearance, necrosis, and invasion of the capsule and strap muscles. Microscopic examination (Figure 1C,D) presented infiltrative atypical spindle cells with moderate pleomorphism arranged in the fascicle with foci of tumor necrosis. The mitotic activity was high (8/10 high-power fields). Lymphatic, vascular, and perineural invasion were observed. Few residual thyroid follicles are seen within fibrosis in the center of the mass. IHC staining (Figure 1) revealed positivity for smooth muscle actin (SMA), a marker typically associated with mesenchymal tissues. The tumor cells were negative for thyroid-specific markers, such as transcription factor 1 (TTF-1), paired-box gene 8 (PAX8), and thyroglobulin, which rules out a thyroid origin. Additionally, the absence of epithelium markers (AE1/AE3) and muscle markers (desmin, smooth muscle heavy chain, caldesmon) further supports the diagnosis of a non-epithelial, non-muscle tumor. These findings are consistent with the diagnosis of primary thyroid fibrosarcoma, a rare type of soft tissue sarcoma.

Unfortunately, positron emission tomography (PET) scan was not performed as it was not reimbursed. The patient underwent postoperative radiotherapy with a total dose of 3000 CGy and received three cycles of chemotherapy with Ifosfamide, which was administered as adjuvant therapy for advanced, unresectable, or metastatic disease [6]. However, no significant response to treatment was observed. At 9 months postdiagnosis, follow-up CT imaging demonstrated progression, with an increase in both the size and number of pulmonary nodules. Consequently, pazopanib, a tyrosine kinase inhibitor, was administered, resulting in a marked reduction in the size of the pulmonary nodules after 2 months (Figure 2C). Despite the initial therapeutic response, disease progression occurred 9 months after starting, marked by the development of a new right neck mass (3 cm) and further enlargement of the pulmonary nodules. FNAB of the right neck mass confirmed recurrent malignant spindle cell neoplasm. The patient passed away 3 months after the recurrence due to respiratory failure.

## 3. Discussion

This case highlights the rarity of primary thyroid fibrosarcoma, a malignancy often mistaken for anaplastic thyroid carcinoma. IHC staining is essential for distinguishing thyroid fibrosarcoma from anaplastic thyroid carcinoma, allowing for an accurate diagnosis and appropriate treatment planning.

Fibrosarcoma is a malignant tumor originating from mesenchymal cells in fibrous connective tissue, primarily composed of aggressive fibroblasts embedded in a collagen matrix. Adult-type fibrosarcoma typically occurs in the collagen-rich connective tissue areas, such as the thighs, knees, arms, and trunk wall [7]. It is less commonly found in the retroperitoneum, and head and neck regions [7]. However, fibrosarcoma of the thyroid gland is exceedingly rare [4], with only 13 cases reported among 142 primary thyroid sarcomas in a 24-year (1990–2014) review [4]. Clinical presentations may include a rapidly growing, painless neck mass, difficulty swallowing, cough, hoarseness, and shortness of breath due to compression and invasion of surrounding structures. Most patients are euthyroid [8]. Imaging findings on both ultrasound and CT are often nonspecific. Ultrasound may reveal areas with mixed echogenicity, ranging from hypo- to hyperechoic [4]. Noncontrast CT often shows inhomogeneous regions with varying densities, while contrast-enhanced CT demonstrates nonhomogenous enhancement [4]. Definitive diagnosis relies on histopathological examination and IHC analysis.

Malignancies histologically similar to anaplastic thyroid cancer but with different treatments and prognoses include poorly differentiated thyroid cancer, medullary thyroid cancer, lymphoma, melanoma, sarcoma, and lung cancer [9, 10]. IHC is essential for accurate differential diagnosis, especially when routine histologic examination cannot distinguish between these malignancies. In thyroid pathology, IHC confirms the cell of origin, particularly in nonfollicular or non-thyroidal lesions. Key markers include thyroglobulin, TTF-1, and PAX8 [11]. Thyroglobulin, the most specific marker of thyroid follicular cells, shows diffuse cytoplasmic staining in well-differentiated thyroid cancers, like papillary and follicular thyroid carcinoma, but is absent in medullary thyroid carcinoma and metastatic tumors to the thyroid. High-grade follicular-derived carcinomas, necrotic tumor foci, and anaplastic thyroid carcinoma may lose thyroglobulin expression. TTF-1 shows nuclear expression in both follicular and parafollicular cells of the thyroid and in lung tissue, widely expressed in differentiated thyroid cancers but retained in less than 20% of anaplastic thyroid cancers. TTF-1 is also expressed in lung adenocarcinomas, a subset of squamous cell carcinoma of pulmonary origin, small cell carcinomas, and neuroendocrine tumors, making it useful for distinguishing primary thyroid tumors from metastatic disease. PAX8, a transcription factor critical for thyroid, kidney, and Müllerian tract development, shows nuclear staining in normal and neoplastic thyrocytes and is retained in most thyroid cancers, including high-grade follicular cell-derived carcinoma and anaplastic carcinoma. Nonfollicular thyroid tumors are usually PAX8-negative. PAX8 is also found in renal, ovarian, and bladder malignancies, and is often used alongside TTF-1 and thyroglobulin to confirm thyroid origin or exclude metastasis from other organs.

Cytokeratins, such as AE1/AE3, CAM5.2, 34BE12, CK18, and CK5/6, are structural proteins in the cytoskeleton of epithelial tissues, with their antibodies serving as valuable tools for distinguishing carcinomas from other tumors. The AE1/AE3 antibody cocktail is widely used in IHC and demonstrates ~80% sensitivity for diagnosing anaplastic thyroid carcinoma [12]. However, the staining intensity and pattern of AE1/AE3 in anaplastic thyroid carcinoma are highly variable. While cytokeratin staining is a useful diagnostic tool, it lacks specificity for anaplastic thyroid carcinoma and requires integration with clinical findings and additional IHC markers to establish a definitive diagnosis [12].

No single IHC marker is diagnostic for fibrosarcoma; instead, a panel of markers is typically used to confirm the diagnosis. The following are some of the most commonly used IHC markers for fibrosarcoma: Vimentin is a general marker of mesenchymal origin that is usually positive in fibrosarcoma. Alpha-SMA (α-SMA) is often positive in fibrosarcoma, as observed in our patient, particularly in areas exhibiting myofibroblastic differentiation. CD34 is expressed by certain fibrosarcomas, particularly those displaying a storiform pattern. CD99 is occasionally positive in fibrosarcoma, though it lacks specificity.

Management of thyroid fibrosarcoma is challenging due to its rarity, with no established treatment guidelines. Therefore, treatment decisions often rely on experienced multidisplinary team. The primary therapy is surgery; however, if the lesion is unresectable or if there has been incomplete resection, adjuvant therapies, such as chemotherapy and/or radiation may be considered. Additionally, systemic therapies, such as tyrosine kinase inhibitor, may be options for patiens who do not respond to chemoradiation or those with distant metastases. Our case report is the first to document the use of pazopanib, a potent and selecctive multitargeted receptor tyrosine kinase inhibitor, specifically for treatment of thyroid fibrosarcoma. Pazopanib works by blocking tumor growth and inhibits angiogenesis. It is approved for the treatment of several advanced cancer, including soft tissue sarcomas [13]. Although, not specifically approved for thyroid fibrosarcoma, pazopanib may be considered as a potential treatment option.

## 4. Conclusions

Thyroid fibrosarcoma, though rare, should be considered in the differential diagnosis of rapidly growing thyroid masses. Accurate diagnosis relies on histopathological evaluation and IHC analysis, which are critical for determining appropriate management strategies.

## Data Availability

The data that support the findings of this study are available from the corresponding author upon reasonable request.
